# *QuickStats*: Age-Adjusted Percentage* of Adults Aged ≥18 Years with Diagnosed Chronic Obstructive Pulmonary Disease,^†^ by Urbanization Level — United States, 2023

**DOI:** 10.15585/mmwr.mm7346a5

**Published:** 2024-11-21

**Figure:**
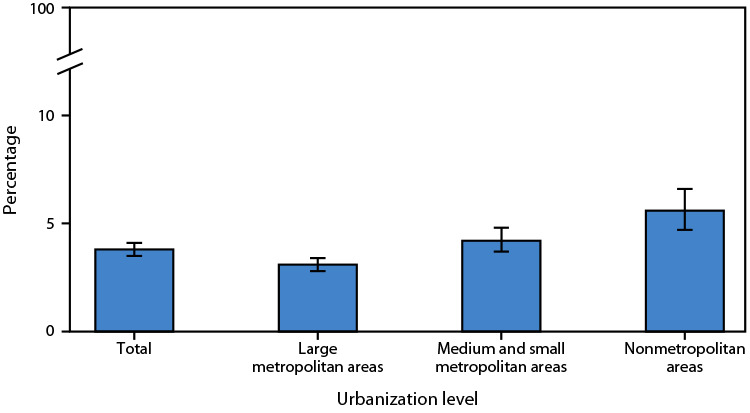
In 2023, the age-adjusted percentage of adults aged ≥18 years with diagnosed chronic obstructive pulmonary disease (COPD) was 3.8%. The prevalence of COPD among adults increased as urbanization level decreased.

**Supplementary Table:** https://stacks.cdc.gov/view/cdc/168877

**Source:** National Center for Health Statistics, National Health Interview Survey, 2023. https://www.cdc.gov/nchs/nhis.htm

For more information on this topic, CDC recommends the following link: https://www.cdc.gov/copd/index.html.

